# Spatial Distribution of *Dermanyssus gallinae* Infestations in Greece and Their Association with Ambient Temperature, Humidity, and Altitude

**DOI:** 10.3390/pathogens13040347

**Published:** 2024-04-22

**Authors:** Georgios Sioutas, Athanasios I. Gelasakis, Elias Papadopoulos

**Affiliations:** 1 Laboratory of Parasitology and Parasitic Diseases, Faculty of Health Sciences, School of Veterinary Medicine, Aristotle University of Thessaloniki, 54124 Thessaloniki, Greece; gsioutas@vet.auth.gr; 2 Laboratory of Anatomy and Physiology of Farm Animals, Department of Animal Science, School of Animal Biosciences, Agricultural University of Athens, 11855 Athens, Greece; gelasakis@aua.gr

**Keywords:** altitude, *Dermanyssus gallinae*, Greece, humidity, laying hens, poultry farms, poultry red mite, prevalence, risk factors, temperature

## Abstract

*Dermanyssus gallinae*, the poultry red mite (PRM), is the most prevalent and harmful ectoparasite of laying hens globally. Although prevalence and risk factor studies can help veterinarians make decisions regarding farm treatments, relevant data are scarce. The present study investigated the prevalence and infestation severity of PRM in poultry farms across Greece and examined potential risk factors. AviVet traps were used to sample 84 farms (51 backyard, 33 industrial) over three years. Farm altitude, temperature, humidity, region, and production systems were assessed as potential risk factors with chi-square tests, initially for all the studied farms and then exclusively for backyard farms. The overall prevalence was 75.0% and was higher in backyard farms (80.4%) compared with industrial ones (66.7%), varying regionally from 66.7 to 90.9%. Altitude and temperature were not significant risk factors, but farms with humidity <60% had a lower infestation risk. Infestation severity did not significantly differ by risk factors. The poultry red mite is highly prevalent across Greek poultry production systems and regions. In the future, global warming, reduced acaricide options, and a ban on cage systems will all threaten a wider spatio-temporal distribution of the PRM, justifying the urgent need for effective monitoring and control methods to protect hen production and welfare and workers’ health.

## 1. Introduction

*Dermanyssus gallinae* (De Geer, 1778), most commonly known as the chicken mite or the poultry red mite (PRM), is undoubtedly the most prevalent and economically deleterious ectoparasite of laying hens worldwide [1,2]. It is highly prevalent in many countries, with the average prevalence in Europe reaching 83%, where it can be found in every farming system, from industrial types (cages, barns) to extensive types (free-range/backyard, organic) [3,4]. Clinical signs in hens include anaemia, stress, irritation, restlessness, immunocompromisation, over-preening, excessive feed conversion ratios, reduced egg production, egg size and quality, blood spots on eggs, and, in severe infestations, even death [1,4]. Moreover, *D. gallinae* can carry or transmit different pathogens, including bacteria such as *Escherichia coli* and *Listeria monocytogenes* [5,6], viruses, i.e., fowlpox virus [7], and even other parasites like *Plasmodium* spp. [8] to both chickens and humans [3]. Poultry red mites are also a concern for public health and have been described as an occupational health hazard for poultry workers [9], although they typically prefer bird to human skin [10,11]. However, the *D. gallinae* that infests pigeons belongs to a different phylogenetic clade (cryptic species) and is much more aggressive towards humans than the PRM that infests chickens [12].

The life cycle of *D. gallinae* can usually be completed within 7 days [13], and it consists of the following five stages: eggs, larvae, protonymphs, deutonymphs, and adults. Larvae have six legs, while nymphs and adults have eight legs. The only non-haematophagous stages are eggs and larvae. Protonymphs and deutonymphs feed to moult to the next stage; female adults feed to lay their eggs, while adult males only feed sporadically [14]. *D. gallinae* is a temporary and obligatory bloodfeeder, preferably feeding during the night for 30 min up to one hour, and then it seeks shelter in cracks and crevices, where it spends the majority of its time [14,15]. *D. gallinae*’s hiding places include areas behind/under cracks, crevices in woods or metal connections, feeders, drinkers, egg conveyor belts, nests, perches, dry manure, and wet litter [1,2,10,14]. Before feeding, mites have a grey colour; after feeding, they have a blood-red colour [14]. Adult PRMs drink 2.7 times their body weight in blood each time, which amounts to 207 μg [10], and a chicken can lose 3% of its blood each night [4]. 

Mites can be transmitted to clean poultry houses in several ways. These include but are not limited to the transfer of infested chickens to clean poultry houses, the use of infested cages and egg cartons, personnel/workers carrying PRMs on their clothing, wild birds (i.e., pigeons) carrying them on their feathers, other animals, both domestic and wild, that may be heavily infested, and by mites migrating at a nearby distance from infested to clean poultry houses [14,16]. In the past, the most common transmission route was by introducing infested chickens [14,16], and in recent years, infested equipment such as cages, crates, and boxes have also been considered important PRM introduction points in industrial poultry operations [17].

There are limited treatments available for the PRM because the mites have developed resistance against many acaricide classes because of the drug residues in eggs and the removal of acaricides from the market [18]. In most European markets like Greece, fluralaner is one of the few compounds licensed for treating PRM infestations [19]. The demand for effective and long-lasting control methods against PRM infestation is urgent and requires a better understanding of its population numbers and survival outside the laboratory under different altitude, temperature, and humidity conditions [1]. Prevalence and risk factor studies can help veterinarians, and poultry farmers make evidence-based decisions about monitoring, infestation risk and treatments considering the spatio-temporal distribution of PRM infestations. Data on *D. gallinae* prevalence in Greece are scarce [20], and very few studies have investigated PRM infestations in relation to farm temperature, humidity, or altitude worldwide [21,22]. Therefore, the aim of the current study was to investigate the prevalence and infestation severity of *D. gallinae* in poultry farms in Greece and examine possible risk factors (altitude, farm type, temperature, and humidity).

## 2. Materials and Methods

### 2.1. Study Area, Meteorological Data, and Altitude Information

Greece covers a total area of 131,957 km^2^ and is located in the southern part of Europe, in the Balkan region, with approximately 80% of its land mass in the mainland with an average altitude of 600 m and the rest of the land shared among its islands. The country has almost 6000 islands and a significant coastline, and it is surrounded by the Eastern Mediterranean, the Ionian, and the Aegean Sea [23,24]. The climate of Greece is characterised as Mediterranean, with warm, dry summers and, usually, long days of sunshine throughout most seasons. From April to September, the mean temperature in southern Greece is around 24 °C, while it is lower in the northern regions. The highest temperatures are recorded between July and August, ranging from 29.0 to 35.0 °C. In contrast, the winters are rainy and relatively mild in most areas, with lower temperatures and heavy snowfalls in the mountains. The coldest months of the year are typically January and February, when the mean minimum temperature often ranges from 5 to 10 °C close to the sea and from 0 to 5 °C across the mainland, while northern Greece usually has even lower temperatures below 0 °C. The average relative humidity (RH) in Greece is between 65% and 75%, with the highest values in December and the lowest in July–August. Furthermore, north-western regions of the country have the highest RH, while southern regions have the lowest RH. Greece’s diverse geography (i.e., large mountain ranges next to lowlands) and dominant weather patterns are responsible for the country’s significant geospatial climatic variations. Because of this, the climate can change from alpine to Mediterranean in just a short distance [23,24]. 

For the current study, meteorological data from the Hellenic National Meteorological Service (HNMS) were used for all the locations where the poultry farms were sampled [24]. The HNMS is the official government organisation that issues Greek weather predictions. For each sampling location and backyard poultry farm, the closest meteorological station was used to extract the mean air temperature and RH values for the sampling month and annually for that year [24]. For industrial poultry farms, in-house temperature and RH measurements were recorded instead since they are closely monitored and remain constant throughout the production cycle regardless of external weather conditions, unlike backyard poultry farms. Geospatial information, including regional altitude for each farm, was collected with an accuracy of around ±1.73 m using NASA’s Shuttle Radar Topography Mission (SRTM) and a Digital Elevation Model (DEM), as showcased in previous studies [25,26]. The precise sampling locations were marked on the map and used to extract the altitude values, measured in meters above sea level (a.s.l.).

### 2.2. AviVet Traps

The AviVet Red Mite Trap™ (Avivet, adVee Dierenartsen, Heeswijk Dinther, The Netherlands) is the evolution of the simple corrugated cardboard trap. It represents a novel, scientifically validated and reliable trap that is simple to use and allows for quantitatively evaluating *D. gallinae* infestation levels in laying hen farms [27]. Each trap consists of a rolled corrugated cardboard piece (thickness of 1 mm, width 60 mm, and length 50 mm) enclosed inside a tylene tube (inner diameter 12 mm, outer diameter 16 mm, and length 50 mm) with a unique serial number for identification. These traps can detect all stages of the PRM life cycle (eggs, larvae, protonymphs, deutonymphs, and adults) [27]. The trap works by forming crevices and cracks inside the rolled cardboard, which are the ideal hiding places for PRMs luring them inside [27].

### 2.3. Dermanyssus gallinae Sampling Method and Duration

The sampling period lasted from April/May to November/December of each year, according to official recommendations for Northern Europe [27], taking into account the climate of Greece and yearly fluctuations in temperatures [24]. Between these months, as the temperature rises, *D. gallinae* becomes active again, starts feeding on chickens, and multiplies rapidly [24,27].

Consequently, live PRM samples were collected with the specialised cardboard traps from May 2021 until December 2023. Farms across all of Greece’s geographical regions—Thrace, Macedonia, Epirus and Ionian Islands, Thessaly, Central Greece, Peloponnese, Crete, and the Aegean Islands—were included in this study. In total, 84 poultry farms were randomly selected and sampled for the presence of *D. gallinae*, consisting of 51 backyard and 33 industrial poultry farms. Each farm had a different infestation history and acaricide treatment employed by the farm owners.

For each poultry farm, 10 Avivet traps were used according to the manufacturer’s recommendations [23], and they were stored inside their numbered plastic sealed bags before sampling. Each trap inside its plastic bag was weighed before sampling using a Highland^®^ Portable Precision Balance-HCB 123 (Adam Equipment Inc., Oxford, CT, USA), which has a capacity of 120 g and a readability of 0.001 g. Afterwards, the trap was removed from its plastic bag and placed in the poultry farm. 

Regarding sampling duration, it has been found that by placing PRM traps in poultry farms for 6–10 days, more mites are collected compared with placing the same traps for two days only [28]. Therefore, in the current study, traps were placed for one week before being collected, as in previous studies [29,30]. 

For sampling locations, the traps were placed horizontally and evenly distributed throughout the whole length, width, and height of the poultry house to cover all potential hiding spots and accurately represent the entire poultry farm. The traps were also positioned outside the reach of chickens, so they did not peck them, and away from air ventilation systems that create strong air currents, which the mites dislike. Preferably, traps were placed in shadows (where mites are likely to be found) and in places where PRMs were more likely to pass by on their way to or returning from a bloodmeal on chickens at night, but not directly on aggregates/mite clusters because mites rarely leave their clusters to enter traps [19,27,31,32,33,34]. Specifically for industrial poultry houses using enriched cages, stacking them on top of each other, and creating multiple floors, the traps were placed on different rows (sides to middle) and floor levels (high, medium, low) at equal distances from one another, beginning from the entryway until the opposite side. Placement spots included perches, particularly the higher ones in aviary systems, under the egg conveyor belt, metal connections, under feeders, under watering lines, and grates/slats in or near nests in backyard poultry farms [5,27,31,35]. The traps were secured using ten black cable ties (thickness of 2 mm, length of 200 mm), which accompanied each set of 10 traps.

The AviVet traps from each farm were gathered after one week, packed again in their individual plastic bags, and shipped to the Laboratory of Parasitology and Parasitic Diseases, School of Veterinary Medicine, Faculty of Health Sciences, Aristotle University of Thessaloniki, Greece. 

### 2.4. Dermanyssus gallinae Identification

Before the second weighing of each plastic bag, the mites in the traps were morphologically examined in order to identify them. For this purpose, a few (2–4) mites from each trap were transferred inside a closed plastic Petri dish and placed in a freezer (−20 °C) for 15 min to reduce their mobility and make them easier to handle. Afterwards, the Petri dish with the mites was placed under a stereomicroscope (Olympus, Research Stereomicroscope System SZH10, PARMA CnS Inc., Tustin, CA, USA), and the mites were identified as *D. gallinae* using available key morphological characteristics for the specific species [36]. Specifically, the identification was based on the characteristic shoulder in the dorsal shield, the epigynal pores in the genitoventral shield, and the placement of setae on the mite [36]. 

### 2.5. Assessment of Infestation Levels

In the laboratory, each trap inside its plastic bag was weighed again using the same scale (Highland^®^ Portable Precision Balance-HCB 123). The difference in the weight of each trap before sampling and after sampling was calculated and marked as X (number ≥0). According to the validation experiments for AviVet by Lammers et al. [27], there is a correlation between the number of *D. gallinae* mites inside each trap and the weight. This relationship is expressed by the regression line Y = 58.50 + 9.56X, where

Y is the total number of *D. gallinae* (eggs, larvae, nymphs, adults) inside each trap;

X is the total weight of *D. gallinae* (eggs, larvae, nymphs, adults) inside each trap.

In order to categorise infestation levels, the average number of *D. gallinae* per trap per farm was calculated based on the average mite mass (weight difference in the trap before placing and after collecting) per farm [33,37] and interpreted as follows:

- Low infestation = 1–500 mites;
- Moderate infestation = 501–2500 mites;
- Heavy/hotspot infestation = >2500 mites.

### 2.6. Statistical Analyses

Mean ± standard deviation and median values, as well as frequencies for the quantitative and qualitative assessment of RPM infestations, respectively, were estimated. Prevalence rates and their 95% confidence intervals (CIs 95%) of *D. gallinae* infestations were calculated per region, as well as for different altitudes (<200 m and ≥200 m a.s.l.), farm types (industrial, backyard), and mean ambient temperature (25–35 °C and <25 °C) and humidity (60–85% and <60%) values. For these estimations, Epitools (ausvet.com.au) and the Wilson score interval method were used. Chi-square tests were performed using SPSS v23 with odds ratios and relative risks being estimated to assess the association among altitude, farm type, and environmental factors (ambient temperature and humidity) and *D. gallinae* infestation status (0 = no infestation, 1 = infestation) and severity (0 = no/low infestation, 1 = moderate/heavy infestation). Farms had a positive infestation status if mites were found in the traps or if farmers had administered fluralaner to the flock (Exzolt^®^, MSD Animal Health, Unterschleißheim, Germany) within three months of sampling, which is the effective duration of the compound and has a sole indication for *D. gallinae*. Statistical significance was set at the 0.05 level.

## 3. Results

The farms sampled had a cumulative production capacity of 1,080,900 laying hens, and in total, 63/84 (75%) poultry farms were positive for *D. gallinae* infestation. The number of tested farms varied by geographical region as follows: in Thrace, 6 farms were tested (all backyard); in Macedonia, 28 farms (11 backyard and 17 industrial); in Epirus and the Ionian islands, 9 farms (5 backyard and 4 industrial); in Thessaly, 9 farms (all backyard); in Central Greece, 17 farms (9 backyard and 8 industrial); in Peloponnese, 4 poultry farms (all industrial), and in Crete and the Aegean islands, 11 farms (all backyard). Taking into account only positive farms, the mean and median numbers of captured mites per positive farm were 2450 ± 3111 and 1072 red mites, respectively (interquartile range = 295–3940 red mites). Table 1 summarises the prevalence rates for the different geographical regions and different altitudes and farm types, as well as the mean ambient temperature and humidity values that were studied.

The chi-square test values, odds ratios, and relative risks for the positivity and severity of *Dermanyssus gallinae* infestations in all the studied farms are summarised in Table 2.

When the mean ambient humidity was favourable (60–85%), farms were more likely to be found positive (ϕ*_c_* = 0.207, and *p* = 0.058) for *D. gallinae* infestations compared with unfavourable (<60%) ambient humidity, with the estimated odds ratio being equal to 2.75 (95% CI, 0.95 to 1.78) and the relative risk increasing by 27%.

Since industrial farms have tightly regulated conditions, the statistical analysis was rerun only for backyard poultry farms because the latter are more exposed to natural environmental conditions (Table 3).

When the mean altitude was < 200 m a.s.l., backyard poultry farms were less likely to have a moderate/heavy infestation (ϕ*_c_* = 0.217, and *p* = 0.121) compared with farms at higher altitudes (≥200 m a.s.l.), with the estimated odds ratio being equal to 0.41 (95% CI, 0.13 to 1.27) and the relative risk decreasing by 34%.

**Figure 1:**
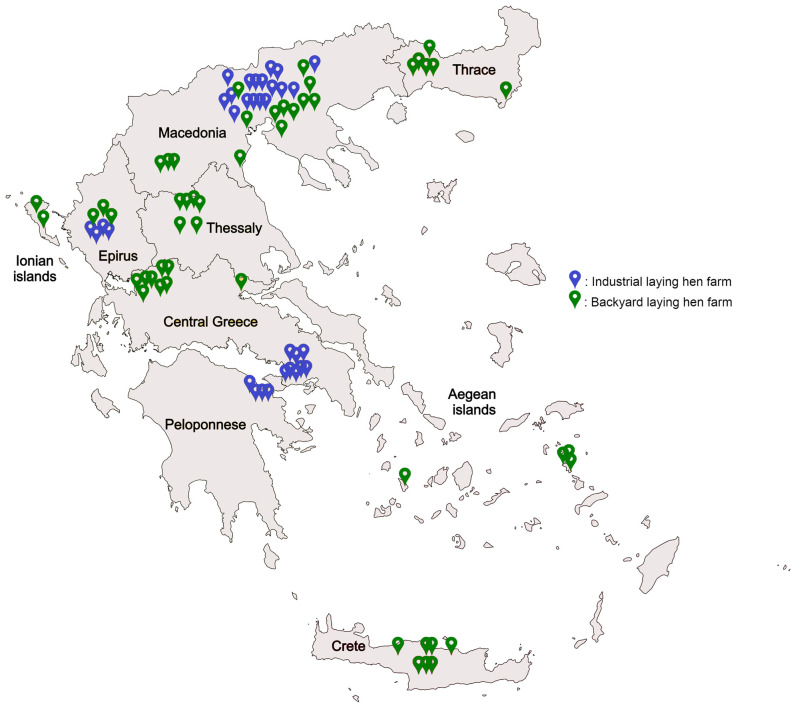
Sampling locations of poultry farms for *Dermanyssus gallinae* in different geographical regions of Greece. Green pins represent the location of backyard laying hen farms that were sampled, and blue pins represent the location of industrial laying hen farms that were sampled.

The sampling locations of poultry farms for *D. gallinae* in each geographical region are illustrated in Figure 1, with different pin colours for industrial and backyard laying hen farms.

## 4. Discussion

This is the first study to investigate the prevalence of *D. gallinae* infestations in poultry farms in Greece at the nationwide level. The total PRM prevalence was 75%, which is lower but still close to the average European prevalence of 83% [3]. Thessaly exhibited the lowest prevalence (66.7%), while Crete and the Aegean islands displayed the highest prevalence (90.9%). It is worth noting that Central Greece and Macedonia produce approximately 64% of the eggs in Greece (41% and 23%, respectively) [37], which explains why the number of sampled farms was higher in these regions. The single similar study in Greece was a small-scale one carried out in 2017 exclusively in Central Macedonia (a subregion of Macedonia), enrolling 12 industrial laying hen farms and demonstrating a PRM prevalence of 100% [20]. In the present study, farms in Macedonia displayed a prevalence of 67.9%, which is still considered high. The observed differences when compared with the study conducted by Arsenopoulos et al. [20] could be associated with the broader sample area (the whole of Macedonia compared with only Central Macedonia), the higher number of the sampled farms (28 vs. 12) and the different types of farms tested (both industrial and backyard compared with only industrial) in our study. Therefore, our study offers updated and more representative information with regard to the prevalence of PRMs in Macedonia.

Crete and the Aegean islands had the highest prevalence in the current study, followed by Thrace, at 90.9% and 83.3%, respectively. Interestingly, only backyard poultry farms were sampled in both regions. The overall prevalence of PRM infestations in backyard poultry farms across the country was 80.4%, and in industrial farms, it was 66.7%, with backyard farms demonstrating a 21% higher infestation risk compared with industrial farms. This is in agreement with previous studies that found backyard poultry farms demonstrate a higher prevalence compared with industrial farms in Sweden [16] and the U.K. [30]. Although both types of poultry farms can be infested with the PRM, extensive/backyard poultry farms offer more hiding spots for the mites, making disinfestation of the premises more difficult and less effective [4,16]. On top of that, hens in extensive/backyard poultry farms can come into contact with wild birds (i.e., pigeons, swallows) that are potential carriers of *D. gallinae*, thus acquiring the infestation [12,14,17,38]. Lastly, industrial/cage poultry farmers tend to employ stricter biosecurity measures that prevent the introduction of *D. gallinae* [4,17]. Regarding infestation severity, there was no statistically significant difference between backyard and industrial poultry farms (*p* = 0.490), indicating that the severity of infestations is not directly linked to farm type.

More than a decade ago, conventional cages were banned in the European Union and replaced with enriched cages, leading many farms to switch to extensive/alternative housing systems [4,39]. This legislation was employed in Sweden many years earlier, in 1999 [16]. Despite improving chicken welfare, banning the traditional cages, in both cases, led to more suitable environments for PRM multiplication, thereby increasing prevalence rates in many countries [4]. The European Union plans to ban all cages by 2027 in the laying hen industry, with some countries (Luxembourg and Austria) already enforcing relevant national legislation towards 100% cage-free poultry farming [40]. The total ban of all cages in the European Union by 2027 is expected to further magnify the problem of *D. gallinae* infestations, increasing its prevalence and spatial distribution. This ban, if implemented, will pose a significant challenge for the Greek laying hen industry, as well, which, according to the most recent official data from the Ministry of Rural Development and Food, has 74.3% of its 5.4 million laying hens housed in enriched cages [41]. This is a slight decrease of 2.2% compared with the percentage of laying hens housed in cages in 2020 (76.5%), but it remains high [40]. 

Regarding location, the altitude where farms were built ranged from 3 m to 871 m a.s.l., and it was investigated as a potential risk factor as it is both directly and indirectly linked to environmental components, as well as the type and diversity of ecosystems (flora and fauna) in the studied farm locations. The notion was that locations at different altitudes, except for different environmental conditions, could also serve as habitats for different potential carriers or natural reservoirs of *D. gallinae*, i.e., rodents and wild birds [17,42,43], that may transmit the ectoparasite to laying hens. However, no significant differences in the occurrence or severity of *D. gallinae* infestation were observed regarding altitude when all the studied farms or only the backyard farms were considered. In another study in neighbouring Turkey, backyard poultry farms located at higher altitudes exhibited higher prevalence rates, although the exact altitude of those regions was not specified, and the higher prevalence rates were likely because of other factors [22]. Furthermore, PRM infestations have been recorded in backyard poultry farms at much higher altitudes than in the current study (i.e., 2880 m a.s.l.), indicating that the PRM survives throughout a wide range of altitudes [44,45,46]. Overall, farm altitude does not seem to be a risk factor per se for *D. gallinae* prevalence or infestation severity, while other altitude-related environmental parameters (i.e., temperatures < 5 °C in mountainous regions) could be more important [22,46].

For temperature, we used ambient temperature in both industrial and backyard poultry farms. However, given that the temperature was controlled in industrial farms, we excluded them and reran the analyses exclusively on the backyard farms. Temperature and humidity remain stable throughout the year for optimal production efficiency in industrial poultry farms, while in backyard poultry farms, temperatures and RH range considerably between months and throughout the year [44]. The mean ambient temperatures ranged from 8.3 °C to 31.2 °C in the current study, but temperature was not a significant risk factor for the prevalence or infestation severity despite this variation. This result agrees with a study in Tunisia, which found no association between temperature and infestation severity in industrial poultry farms [47]. To understand why temperature is not a significant risk factor, it is worth considering the wide range of temperatures in which *D. gallinae* can complete its life cycle, as analysed below.

Different laboratory studies have resulted in relatively similar conditions regarding the optimal temperature and RH for *D. gallinae* to complete its life cycle in the shortest time possible. Furthermore, each life stage of the mite seems to prefer slightly different temperature and RH values than the other stages. Male adults seek to reproduce on and off the host, while females search for a bloodmeal to lay their eggs. Oviposition starts after feeding in 12–24 h at 20–35 °C and 65–85% RH, or in 86 h at 15 °C, with females laying one to nine eggs (typically four to seven) [1,10,14,15,29]. They can lay eggs at a wide range of temperatures, from 5 to 45 °C, with the most suitable being at 20.0–28.6 °C [1,48]. In addition, females require a bloodmeal before each oviposition [10,14,15], usually having eight ovipositions and 30 eggs in total, but they only require to reproduce once to lay fertile eggs [14,15,49]. At 20–30 °C and 60–90% RH, eggs hatch in 24–48 h, and the larvae moult into protonymphs in approximately 24 h [1,10,14,29,48]. The total time required for the eggs to hatch into larvae and then moult into protonymphs is at least 48 h [10,13,48]. Although eggs are quite resistant, they cannot tolerate desiccation [29]. Thereafter, the protonymph feeds on blood and moults into a deutonymph in around 24 h at 25–35 °C and 65–85% RH [14,29,48]. A slightly high optimal temperature compared with eggs and larvae is also required for the deutonymph to become an adult, taking 27 h at 30 °C and 70–85% RH [29]. The life cycle can be completed in 6–7 days at 25–35 °C and 70–85% RH [13,29,50] with the optimal temperature at 30 °C [29] or in 8–9 days at 25.0–28.3 °C and 60–75% RH [10,48]. Notably, when the temperature drops to 15 °C, the life cycle takes 4 weeks [29], further explaining why the mites do not increase as rapidly during the autumn/winter. Remarkably, the progeny of just one female can be more than 2600 mites in 8 weeks, doubling their numbers every few days. Mite populations increase for 4–6 months before plateauing, potentially reaching 200,000 per hen and millions in total, which explains their exponential growth under farm conditions [14,21,48,51,52]. Depending on the environmental conditions, 2–4 generations can occur in a month and 15–50 generations in a year [53]. 

Concerning viability and longevity, the mites are quite persistent and can survive under variable natural farm conditions, i.e., at 25 °C and 80% RH or at 5 °C and 29% RH for 8.5–9 months [1,50,54]. Therefore, in contrast to previous suggestions [14], keeping poultry houses empty for 4–5 months between flocks is insufficient to eradicate the PRM. In addition, all PRM stages are susceptible to desiccation, while all stages perform better at humidity values varying from 70 to 90% RH, although entomopathogenic fungi may kill some mites at such high humidity [1]. Lethal temperatures for the mites are −20 °C for 20 min and 45 °C for 2 h [1], with all the stages starting to exhibit low viability at temperatures ≥35 °C because of heat stress [29]. Eggs do not hatch at winter temperatures < 5 °C or during the summer at >40 °C; thus, the life cycle stops [1,48,55]. Lethal temperatures (i.e., 45 °C) can only be achieved during the empty period, but even then, heating can melt plastic waterers, feeders, and other heat-sensitive equipment in the poultry house or even start fires [56]. Therefore, in the hot Greek summer, when temperatures are approximately 30 °C, provided that the climate is not too dry, the conditions, particularly for backyard farms, are ideal for the PRM to complete its life cycle as quickly as possible in less than a week [29] and reach high prevalence as demonstrated in previous studies [57]. In certain months, like July and August, PRM numbers might plateau in areas with temperatures above ≥35 °C [23,24]. 

Based on the above studies, it is also evident that the farm temperatures in the current study (8.3 °C to 31.2 °C) were inside the range in which *D. gallinae* can survive (−20–45 °C) and also inside the range in which the eggs hatch (5–40 °C). Although the farm temperatures were not always ideal (25–35 °C) for the red mite, they still allowed for the life cycle to be completed. The optimal farm temperature employed by many industrial poultry farmers to maintain high egg production is between 19 °C and 22 °C [58], which is even narrower compared with the temperature where the PRM thrives [48]. Although temperatures in laboratory studies can range from −20 °C to 65 °C to kill PRMs [1], this is not possible under farm conditions with hens present. 

In contrast to temperature, ambient humidity in the present study ranged from 41.8% to 81.1% RH, which is much wider than the range where the PRM thrives [1,29,59]. Laying hens can have a high egg production between a broad range of 25% and 75% RH [60], performing even better at low (25%) to medium (50%) RH [60]. On the other hand, the PRM struggles under such low RH because, as mentioned earlier, all stages are sensitive to dehydration [1,29,59]. Our findings agree with the above studies because although RH was not a significant risk factor for the prevalence or infestation severity, poultry houses with <60% RH had a 27% lower infestation risk than poultry houses with 60–85% RH. This result is quite important, especially for industrial laying farms, because although reaching the extreme RH values (4.3% and 90%) [1] employed in PRM laboratory studies is difficult, lowering the RH in a poultry house below 60% through the use of fans, ventilation systems, or moisture-proof materials is achievable. Thus, a lower RH inside a poultry farm may lead to a decreased infestation risk or slow the proliferation of the mites. This information can be indirectly applied by backyard poultry farmers as well, by timing the treatment when the environmental RH is low enough so that the PRM population is suppressed even lower for longer, thus making treatment more effective. Alternatively, poultry farmers can anticipate a rapid increase in PRM numbers when the RH rises above 60%. It is also important to assess the efficacy of each treatment against the PRM in both high and low RH since resistance reports of PRMs against chemical agents are increasing throughout the world [61,62,63]. Assessing the emergence of resistance is essential in implementing any Integrated Pest Management (IPM) strategy against the PRM [64].

Nevertheless, in the following years, because of extensive global climate change, it is expected that the climate in Greece will become hotter and dryer and will have more severe thunderstorms [23,24]. This trend and the gradual increase in mean temperatures and lower RH across Greece [65,66] are expected to impact the PRM in different ways. Firstly, it will provide more favourable conditions for the survival and reproduction of the PRM, particularly during winter months (December–February) when the temperatures are the lowest and the RH is the highest [23,24]. Until now, many Greek regions had temperatures ≤5 °C during the winter months, which stopped PRM eggs from hatching [1], reduced the mites’ activity [14], and slowed their proliferation [67]. A slight increase in temperatures above 5 °C during these months could allow the PRM to complete its life cycle throughout the year, posing an even bigger problem for poultry farmers who already have limited choices for treatment [18]. On the contrary, a slight decrease in RH during winter will not severely affect *D. gallinae*’s multiplication since the RH is well above 60% in that season. The effects of climate change are already visible in Greece because, in the last year of sampling of the present study (2023), an infested backyard poultry farm was found at the end of December in an area in Central Greece with a temperature of 8.3 °C and an RH of 65.8%. Infestations so late in the year were not recorded in the first two years of sampling (2021 and 2022), thus indicating that in the near future, the PRM might be a problem throughout the year. It is also worth noting that, as proven in laboratory experiments, mites are extremely sensitive to temperature changes, and heat can act as a powerful stimulus, activating mites to seek a bloodmeal [2,68].

The second significant impact climate change is expected to have on PRM prevalence in Greece is during summer. An increase in mean temperatures [65,66] will help *D. gallinae* proliferate, except possibly during the warmest months of July and August, where temperatures are already between 29.0 and 35.0 °C and the RH is the lowest [23,24]. If temperatures exceed 35.0 °C, with even dryer conditions, *D. gallinae* will be negatively affected by heat stress [26] and dehydration [1,26], potentially slowing its proliferation in those months. Nonetheless, global warming and extreme weather events are overall expected to facilitate *D. gallinae*’s geospatial expansion, like the unprecedented 2003 summer heatwave that caused the mortality of many chickens following a rapid increase in PRM numbers [69]. 

Lastly, concerning infestation severity, it has been found that 99.6% of the variation in the weight of each trap can be attributed to the variation in the total number of *D. gallinae* [27]. In other words, the weight of *D. gallinae* is a very strong predictor of the total number of *D. gallinae* in a trap. Additionally, the total number of *D. gallinae* in the AviVet traps had a strong correlation (93.8%) with the total number of *D. gallinae* in the sampled cages under experimental conditions, which is the highest among the ones reported for any PRM monitoring method until now [27]. Since it is impossible to accurately locate and count all *D. gallinae* that infest a poultry house under field conditions and none of the available methods is perfect [27,33], the AviVet trap is top-ranked as the most sensitive, reliable method to estimate *D. gallinae* population numbers and infestation levels (low, moderate, heavy) in an infested poultry house [27,33]. Regarding specificity, although the AviVet traps are proven to be specific for *D. gallinae* under experimental conditions [27], in the current research study, some spiders and feather lice were rarely identified inside the plastic bags that were easily noticed macroscopically and removed before weighing.

In previous studies, high mite infestations with AviVet or similar cardboard traps have also been recorded, with mite counts per trap even reaching 81,000 mites [70], 20,000 mites [27], 25,000 mites [21], 8300 mites [71], or 18,223 mites [72]. In the current study, the average mite numbers per positive farm ranged from 59 to 13,199. Similar to other ectoparasites, *D. gallinae* exhibits high spatial aggregation in poultry farms, meaning there is an uneven distribution of mites, with some hiding spots/traps and farms having many mites and some having only a few mites [70,73]. This phenomenon can be minimised at the farm level by using 10 traps spread throughout the poultry farm and obtaining a representative number of mites under a setup similar to the one used in the present study [73]. Nonetheless, given that the mean number of mites per positive farm was 2450 ± 3111, displaying a positive skew distribution, the median provided a more accurate representation of the central value. As expected, the median was lower at 1072 red mites (interquartile range = 295–3940 red mites), indicating that only a few positive farms had heavy infestations, with most positive farms demonstrating low to moderate infestations.

## 5. Conclusions

A high prevalence rate of *D. gallinae* in both backyard and industrial poultry farms in Greece was evidenced. Altitude, temperature, and humidity were not identified as significant risk factors for PRM infestation and severity when all the studied farms or exclusively the backyard farms were considered, although farms with low humidity (<60% RH) had a clear tendency towards a lower infestation risk. Global warming and dryer climates, the potential ban of all cages in poultry farming, and the scarcity of licensed treatments for the PRM are expected to increase *D. gallinae* prevalence and its spatial distribution in Greece and the rest of Europe in the future. Therefore, proper monitoring techniques, strict and efficient hygiene measures, and novel therapies are necessary to improve the welfare of laying hens, mitigate the negative effects on their productivity, and minimise the exposure of humans to PRM, which could also challenge their health. 

## Figures and Tables

**Table 1:** *Dermanyssus gallinae* prevalence rates for different Greek geographical regions, as well as for different altitudes, farm types, and mean ambient temperature and humidity values studied.

Risk Factor	Risk Factor Group	Number of Tested Farms	Prevalence (%)	95% CI of Prevalence (%)
Region	Thrace	6	83.3	43.7–97.0
	Macedonia	28	67.9	49.3–82.1
	Epirus and Ionian islands	9	77.8	45.3–93.7
	Thessaly	9	66.7	35.4–87.9
	Central Greece	17	76.5	52.7–90.4
	Peloponnese	4	75.0	30.1–95.4
	Crete and Aegean islands	11	90.9	62.3–98.4
Altitude	<200 m a.s.l.	48	72.9	59.0–83.4
	≥200 m a.s.l.	36	77.8	61.9–88.3
Farm type	Backyard	51	80.4	67.5–89.0
	Industrial	33	66.7	49.6–80.3
Mean ambient temperature	25–35 °C	48	70.8	56.8–81.8
	<25 °C	36	80.6	65.0–90.3
Mean ambient humidity	60–85%	39	84.6	70.3–92.8
	<60%	45	66.7	52.1–78.6
Total Prevalence	-	84	75.0	64.8–83.0

CI: confidence interval; a.s.l.: above sea level.

**Table 2:** Chi-square test values, odds ratios, and relative risks for positivity and severity of *Dermanyssus gallinae* infestations in all the studied farms.

**Positivity**									
**Variable**	**Group**	**% Negative** **(25.0%, *n* = 21)**	**% Positive** **(75.0%, *n* = 63)**	***χ* ^2^**	**df**	***p*-Value**	**ϕ*_c_***	**OR (95% CI)**	***RR***
Altitude	-	-	-	0.26	1	0.611	0.056	0.77 (0.28–2.11)	0.94
	<200 m a.s.l.	61.9 (13)	55.6 (35)						
	≥200 m a.s.l. *	38.1 (8)	44.4 (28)						
Farming system	-	-	-	2.01	1	0.156	0.155	2.05 (0.75–5.59)	1.21
	Backyard	47.6 (10)	65.1 (41)						
	Industrial *	52.4 (11)	34.9 (22)						
Mean ambient temperature	-	-	-	1.04	1	0.309	0.111	0.59 (0.21–1.65)	0.88
	25–35 °C	66.7 (14)	54.0 (34)						
	<25 °C *	33.3 (7)	46.0 (29)						
Mean ambient humidity	-	-	-	3.59	1	0.058	0.207	2.75 (0.95–8.00)	1.27
	60–85%	28.6 (6)	52.4 (33)						
	<60% *	71.4 (15)	47.6 (30)						
**Severity**									
**Variable**	**Group**	**% No/** **Low Infestation** **(44.0%, *n* = 37)**	**% Moderate/** **Heavy Infestation** **(56.0%, *n* = 47)**	***χ* ^2^**	**df**	***p*-Value**	**ϕ*_c_***	**OR (95% CI)**	***RR***
Altitude	-	-	-	0.68	1	0.409	0.090	0.69 (0.29–1.66)	0.85
	<200 m a.s.l.	62.2 (23)	53.2 (25)						
	≥200 m a.s.l. *	37.8 (14)	46.8 (22)						
Farming system	-	-	-	0.48	1	0.490	0.075	0.73 (0.30–1.78)	0.87
	Backyard	64.9 (24)	57.4 (27)						
	Industrial *	35.1 (13)	42.6 (20)						
Mean ambient temperature	-	-	-	0.15	1	0.703	0.042	0.84 (0.35–2.02)	0.93
	25–35 °C	59.5 (22)	55.3 (26)						
	<25 °C *	40.5 (15)	44.7 (21)						
Mean ambient humidity	-	-	-	0.13	1	0.717	0.039	0.85 (0.36–2.02)	0.93
	60–85%	48.6 (18)	44.7 (21)						
	<60% *	51.4 (19)	55.3 (26)						

* Reference groups, df: degrees of freedom, ϕ*_c_*: Cramér’s V, OR: odds ratio, RR: relative risk.

**Table 3:** Chi-square test values, odds ratios, and relative risks for positivity and severity of *Dermanyssus gallinae* infestations, exclusively in backyard farms.

**Positivity**									
**Variable**	**Group**	**%** **Negative** **(25.0%, *n* = 10)**	**%** **Positive** **(75.0%, *n* = 41)**	***χ* ^2^**	**df**	***p*-Value**	**ϕ*_c_***	**OR (95% CI)**	***RR***
Altitude	-	-	-	0.01	1	0.945	0.010	1.05 (0.26–4.18)	1.01
	<200 m a.s.l.	50.0 (5)	51.2 (21)						
	≥200 m a.s.l. *	50.0 (5)	48.8 (20)						
Mean ambient temperature	-	-	-	1.80	1	0.180	0.188	0.37 (0.08–1.63)	0.83
	25–35 °C	70.0 (7)	46.3 (19)						
	<25 °C *	30.0 (3)	53.7 (22)						
Mean ambient humidity	-	-	-	2.25	1	0.133	0.210	2.89 (0.70–11.90)	1.24
	60–85%	40.0 (4)	65.9 (27)						
	<60% *	60.0 (6)	34.1 (14)						
**Severity**									
**Variable**	**Group**	**% No/** **Low Infestation** **(47.1%, *n* = 24)**	**% Moderate/** **Heavy Infestation** **(52.9%, *n* = 27)**	***χ* ^2^**	**df**	***p*-Value**	**ϕ*_c_***	**OR (95% CI)**	***RR***
Altitude	-	-	-	2.41	1	0.121	0.217	0.41 (0.13–1.27)	0.66
	<200 m a.s.l.	62.5 (15)	40.7 (11)						
	≥200 m a.s.l. *	37.5 (9)	59.3 (16)						
Mean ambient temperature	-	-	-	0.18	1	0.668	0.060	0.79 (0.26–2.36)	0.89
	25–35 °C	54.2 (13)	48.1 (13)						
	<25 °C *	45.8 (11)	51.9 (14)						
Mean ambient humidity	-	-	-	0.66	1	0.417	0.114	0.63 (0.20–1.95)	0.81
	60–85%	66.7 (16)	55.6 (15)						
	<60% *	33.3 (8)	44.4 (12)						

* Reference groups, df: degrees of freedom, ϕ*_c_*: Cramér’s V, OR: odds ratio, RR: relative risk.

## Data Availability

The data presented in this study are available in this article.
